# 4-[(*Z*)-(2-Fur­yl)(2-naphthyl­amino)methyl­ene)]-3-methyl-1-phenyl-1*H*-pyrazol-5(4*H*)-one

**DOI:** 10.1107/S1600536809025586

**Published:** 2009-07-11

**Authors:** Jing Li, Jin-Zhou Li, Jing-Qi Li, Heng-Qiang Zhang, Jia-Min Li

**Affiliations:** a College of Chemistry and Chemical Engineering, Harbin Normal University, Harbin 150025, People’s Republic of China

## Abstract

The title compound, C_25_H_19_N_3_O_2_, crystallizes as discrete mol­ecules which are well ordered through one intra­molecular N—H⋯O hydrogen bond. Structural analysis indicates that the mol­ecules exist as the amine–one form.

## Related literature

For 4-heterocyclic acyl­pyrazolones, see: Dong *et al.* (1983 ▶). For 4-heterocyclic acyl­pyrazolones derivatives as NMR shift-reagents, see: Mehrotra *et al.* (1978 ▶). For their pharmacological and physiological activity, see: Li *et al.* (2000 ▶). For related structures, see: Uzoukwu *et al.*(1993 ▶); Holzer *et al.* (1999 ▶); Peng *et al.* (2004 ▶); Chai *et al.* (2005 ▶); Lü *et al.* (2006 ▶); Arıcı *et al.* (1999 ▶). For the synthesis, see: Jensen (1959 ▶). 
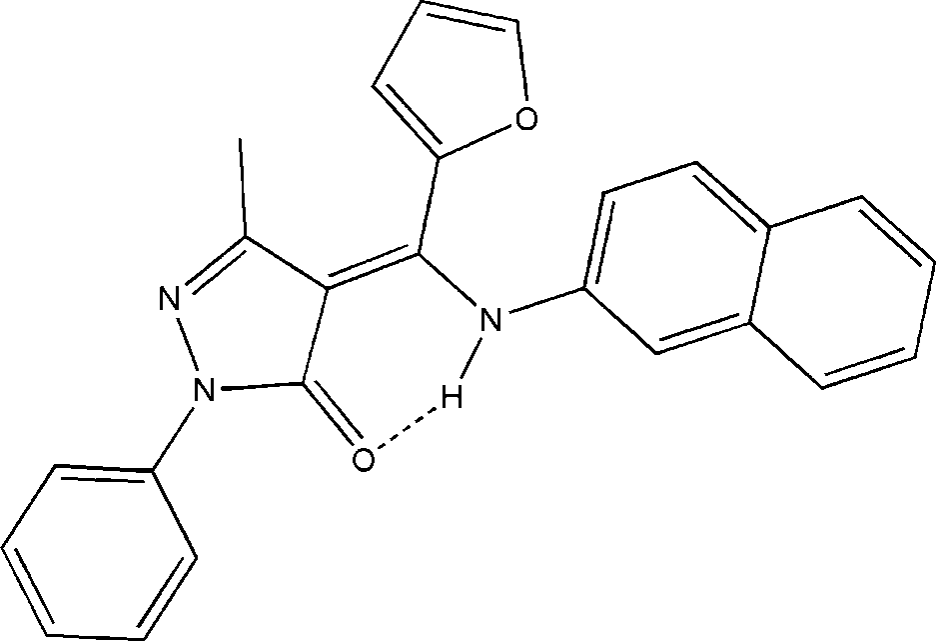


## Experimental

### 

#### Crystal data

C_25_H_19_N_3_O_2_
*M* *_r_* = 393.43
Monoclinic, 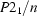

*a* = 9.8484 (10) Å
*b* = 17.5071 (18) Å
*c* = 12.1549 (13) Å
β = 108.836 (2)°
*V* = 1983.5 (4) Å^3^
*Z* = 4
Mo *K*α radiation
μ = 0.09 mm^−1^
*T* = 295 K
0.44 × 0.30 × 0.20 mm

#### Data collection

Bruker SMART CCD area-detector diffractometer
Absorption correction: multi-scan (*SADABS*; Sheldrick, 2005 ▶) *T*_min_ = 0.959, *T*_max_ = 0.983
13952 measured reflections
4755 independent reflections
3166 reflections with *I* > 2σ(*I*)
*R*_int_ = 0.026

#### Refinement

*R*[*F* ^2^ > 2σ(*F* ^2^)] = 0.045
*wR*(*F* ^2^) = 0.120
*S* = 1.05
4755 reflections
276 parameters
H atoms treated by a mixture of independent and constrained refinement
Δρ_max_ = 0.16 e Å^−3^
Δρ_min_ = −0.18 e Å^−3^

### 

Data collection: *SMART* (Bruker, 2005 ▶); cell refinement: *SAINT* (Bruker, 2005 ▶); data reduction: *SAINT*; program(s) used to solve structure: *SHELXS97* (Sheldrick, 2008 ▶); program(s) used to refine structure: *SHELXL97* (Sheldrick, 2008 ▶); molecular graphics: *SHELXTL* (Sheldrick, 2008 ▶); software used to prepare material for publication: *SHELXL97*.

## Supplementary Material

Crystal structure: contains datablocks I, global. DOI: 10.1107/S1600536809025586/hg2530sup1.cif

Structure factors: contains datablocks I. DOI: 10.1107/S1600536809025586/hg2530Isup2.hkl

Additional supplementary materials:  crystallographic information; 3D view; checkCIF report

## Figures and Tables

**Table 1:** Hydrogen-bond geometry (Å, °)

*D*—H⋯*A*	*D*—H	H⋯*A*	*D*⋯*A*	*D*—H⋯*A*
N3—H3*A*⋯O1	0.890 (17)	1.930 (17)	2.7030 (17)	144.3 (16)

**Fig. 1.**
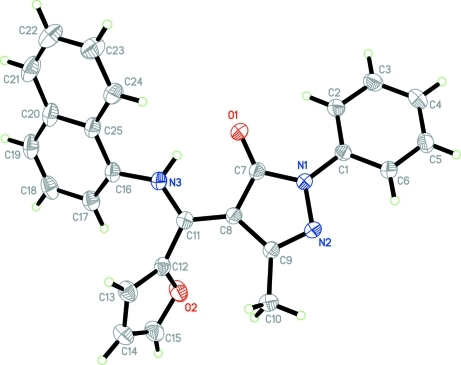
The molecular structure of (I) (thermal ellipsoids are shown at 30% probability levels).

## supplementary crystallographic information

### Comment

1-phenyl-3-methyl-4-(2-furoyl)-5-pyrazolone (HPMαFP), is a member of a family of 4-heterocyclic acylpyrazolones, first synthesized in 1983 (Dong *et al.*, 1983). Such 4-heterocyclic acylpyrazolones derivatives was found to be useful as NMR shift-reagents (Mehrotra *et al.*, 1978), and it is also important in understanding the behaviour of these compounds with respect to the mechanisms of pharmacological activities and physiological activities (Li *et al.*, 2000). As part of this work, we synthesized the title compound (I) derived from HPMαFP, and its structure is reported here.

The molecular structure of (I) is shown in Fig. 1. The C(7)–O(1) distance is 1.248 (3)Å which is  shorter than that for C–OH in some pyrazolone compounds 1.319 (5)Å (Uzoukwu *et al.*, 1993) and 1.313 (2)Å (Holzer *et al.*, 1999), whereas it is close to the distances for C=O in similar compounds: 1.256 (3)Å (Uzoukwu *et al.*,1993) and 1.254 (2) Å (Peng *et al.*, 2004). The C(8)–C(11)(1.392 (8)Å) is shorter than the C–C (1.53 Å), but close to C–C in some correlative compounds,1.400 (4) Å (Chai *et al.*,  2005). The C(11)–N(3) distances is 1.338 (8) Å, which is longer than the values of 1.292 Å (Peng *et al.*, 2004) and 1.318 (3) Å (Lü *et al.*, 2006) for C=N inpyrazolone compounds, but similar to that for C–N (1.339 Å) (Arıcı *et al.*, 1999). So we conclude that the title compound exist in the keto-form in solid state.  The N–H proton is strongly hydrogen bonded with the O(1) atom, the N(3)···O(1) distances is 2.7030 (2)Å and the angle of N(3)–H(3)···O(1) is 144.3 (16)°. Therefore, the crystal structure study shows that the compound exists in the amine-one form.

### Experimental

All reagents were obtained from commercial sources and used without further purification. HPMαFP was synthesized according to the method proposed by Jensen (1959). A mixture of a 10 ml HPMαFP (1 mmol, 0.2683 g) anhydrous ethanol solution and 10 mlα-naphthylamine (1 mmol, 0.1432 g) ethanol solution was refluxed for 4–5 h at 75–80°C, a deep-yellow product which was precipitated, filtered off and washed with anhydrous ethanol for several times, dried in air. The deep-yellow powder was recrystallized from ethanol and the single crystals were obtained at room temperature after 3 days.

### Refinement

The H atom bonded to N3 was located in a difference map and refined freely. Other H atoms were placed in calculated positions, with C—H = 0.93 for phenyl, furyl and naphthyl0.96 for methyl H atoms,and refined as riding, with *U*_iso_(H) = 1.2*U*_eq_ (C) for phenyl and naphthyl H, and 1.5_eq_U(C) for methyl H.

### Crystal data

**Table:**

C_25_H_19_N_3_O_2_	*F*(000) = 824.0
*M**_r_* = 393.43	*D*_x_ = 1.317 Mg m^−^^3^
Monoclinic, *P*2_1_/*n*	Mo *K*α radiation, λ = 0.71073 Å
Hall symbol: -P 2yn	Cell parameters from 3130 reflections
*a* = 9.8484 (10) Å	θ = 2.6–28.0°
*b* = 17.5071 (18) Å	µ = 0.09 mm^−^^1^
*c* = 12.1549 (13) Å	*T* = 295 K
β = 108.836 (2)°	Block, yellow
*V* = 1983.5 (4) Å^3^	0.44 × 0.30 × 0.20 mm
*Z* = 4	

### Data collection

**Table:**

Bruker SMART CCD area-detector diffractometer	4755 independent reflections
Radiation source: fine-focus sealed tube	3166 reflections with *I* > 2σ(*I*)
graphite	*R*_int_ = 0.026
φ and ω scans	θ_max_ = 28.0°, θ_min_ = 2.6°
Absorption correction: multi-scan (*SADABS*; Sheldrick, 2005)	*h* = −12→12
*T*_min_ = 0.959, *T*_max_ = 0.983	*k* = −23→21
13952 measured reflections	*l* = −16→15

### Refinement

**Table:**

Refinement on *F*^2^	Primary atom site location: structure-invariant direct methods
Least-squares matrix: full	Secondary atom site location: difference Fourier map
*R*[*F*^2^ > 2σ(*F*^2^)] = 0.045	Hydrogen site location: inferred from neighbouring sites
*wR*(*F*^2^) = 0.120	H atoms treated by a mixture of independent and constrained refinement
*S* = 1.05	*w* = 1/[σ^2^(*F*_o_^2^) + (0.05*P*)^2^ + 0.2364*P*] where *P* = (*F*_o_^2^ + 2*F*_c_^2^)/3
4755 reflections	(Δ/σ)_max_ = 0.001
276 parameters	Δρ_max_ = 0.16 e Å^−^^3^
0 restraints	Δρ_min_ = −0.18 e Å^−^^3^

### Special details

**Table:**

Geometry. All e.s.d.'s (except the e.s.d. in the dihedral angle between two l.s. planes) are estimated using the full covariance matrix. The cell e.s.d.'s are taken into account individually in the estimation of e.s.d.'s in distances, angles and torsion angles; correlations between e.s.d.'s in cell parameters are only used when they are defined by crystal symmetry. An approximate (isotropic) treatment of cell e.s.d.'s is used for estimating e.s.d.'s involving l.s. planes.
Refinement. Refinement of *F*^2^ against ALL reflections. The weighted *R*-factor *wR* and goodness of fit *S* are based on *F*^2^, conventional *R*-factors *R* are based on *F*, with *F* set to zero for negative *F*^2^. The threshold expression of *F*^2^ > σ(*F*^2^) is used only for calculating *R*-factors(gt) *etc*. and is not relevant to the choice of reflections for refinement. *R*-factors based on *F*^2^ are statistically about twice as large as those based on *F*, and *R*- factors based on ALL data will be even larger.

### Fractional atomic coordinates and isotropic or equivalent isotropic displacement parameters (Å^2^)

**Table:**

	*x*	*y*	*z*	*U*_iso_*/*U*_eq_	
O1	0.31096 (12)	0.99855 (6)	0.59990 (9)	0.0549 (3)	
N2	0.45802 (13)	0.85606 (7)	0.46997 (10)	0.0432 (3)	
N1	0.34379 (12)	0.90162 (7)	0.47756 (10)	0.0399 (3)	
N3	0.55828 (15)	1.02258 (7)	0.77544 (11)	0.0465 (3)	
O2	0.75042 (12)	0.85524 (6)	0.83717 (10)	0.0592 (3)	
C1	0.20702 (15)	0.89273 (8)	0.39232 (12)	0.0390 (3)	
C11	0.61359 (16)	0.96190 (8)	0.73800 (12)	0.0397 (3)	
C7	0.38634 (16)	0.94942 (8)	0.57273 (12)	0.0406 (3)	
C16	0.59012 (16)	1.05724 (9)	0.88701 (13)	0.0449 (4)	
C9	0.56923 (16)	0.87256 (8)	0.56016 (12)	0.0409 (3)	
C8	0.53422 (15)	0.92941 (8)	0.63218 (12)	0.0394 (3)	
C12	0.75221 (16)	0.93050 (8)	0.80752 (12)	0.0418 (3)	
C6	0.19138 (17)	0.84437 (9)	0.29868 (13)	0.0457 (4)	
H6	0.2712	0.8203	0.2895	0.055*	
C25	0.53169 (17)	1.13195 (9)	0.88745 (13)	0.0469 (4)	
C17	0.65935 (18)	1.02042 (10)	0.98919 (14)	0.0535 (4)	
H17	0.6968	0.9718	0.9881	0.064*	
C2	0.08712 (17)	0.92954 (9)	0.40357 (14)	0.0490 (4)	
H2	0.0965	0.9628	0.4653	0.059*	
C4	−0.06237 (18)	0.86776 (10)	0.23023 (15)	0.0564 (4)	
H4	−0.1525	0.8592	0.1763	0.068*	
C10	0.71017 (17)	0.83648 (10)	0.57112 (15)	0.0563 (4)	
H10A	0.7012	0.8060	0.5035	0.085*	
H10B	0.7393	0.8047	0.6391	0.085*	
H10C	0.7808	0.8756	0.5778	0.085*	
C18	0.67368 (19)	1.05648 (12)	1.09619 (14)	0.0628 (5)	
H18	0.7246	1.0322	1.1653	0.075*	
C24	0.46253 (18)	1.17379 (10)	0.78515 (15)	0.0546 (4)	
H24	0.4562	1.1530	0.7133	0.065*	
C5	0.05709 (18)	0.83200 (10)	0.21910 (14)	0.0534 (4)	
H5	0.0471	0.7990	0.1570	0.064*	
C3	−0.04613 (18)	0.91643 (10)	0.32260 (15)	0.0564 (4)	
H3	−0.1262	0.9409	0.3306	0.068*	
C19	0.6143 (2)	1.12593 (12)	1.09975 (15)	0.0645 (5)	
H19	0.6215	1.1477	1.1712	0.077*	
C13	0.88543 (18)	0.95840 (10)	0.85144 (14)	0.0545 (4)	
H13	0.9150	1.0081	0.8444	0.065*	
C21	0.4782 (2)	1.23835 (12)	0.99697 (19)	0.0748 (6)	
H21	0.4819	1.2605	1.0675	0.090*	
C20	0.54195 (19)	1.16567 (10)	0.99658 (15)	0.0561 (4)	
C15	0.8882 (2)	0.83722 (11)	0.90023 (15)	0.0651 (5)	
H15	0.9184	0.7893	0.9315	0.078*	
C14	0.97269 (19)	0.89690 (11)	0.91091 (16)	0.0624 (5)	
H14	1.0709	0.8985	0.9500	0.075*	
C23	0.4050 (2)	1.24393 (11)	0.78978 (18)	0.0689 (5)	
H23	0.3609	1.2706	0.7214	0.083*	
C22	0.4119 (3)	1.27615 (12)	0.8966 (2)	0.0804 (6)	
H22	0.3708	1.3237	0.8988	0.096*	
H3A	0.4738 (19)	1.0356 (10)	0.7248 (15)	0.058 (5)*	

### Atomic displacement parameters (Å^2^)

**Table:**

	*U*^11^	*U*^22^	*U*^33^	*U*^12^	*U*^13^	*U*^23^
O1	0.0514 (7)	0.0583 (7)	0.0513 (7)	0.0136 (5)	0.0115 (5)	−0.0159 (5)
N2	0.0433 (7)	0.0436 (7)	0.0423 (7)	0.0067 (5)	0.0134 (6)	−0.0054 (5)
N1	0.0391 (7)	0.0411 (7)	0.0383 (6)	0.0041 (5)	0.0110 (5)	−0.0056 (5)
N3	0.0498 (8)	0.0483 (8)	0.0369 (7)	0.0048 (6)	0.0076 (6)	−0.0075 (6)
O2	0.0605 (7)	0.0436 (6)	0.0581 (7)	−0.0067 (5)	−0.0022 (6)	0.0069 (5)
C1	0.0409 (8)	0.0386 (8)	0.0364 (7)	0.0015 (6)	0.0110 (6)	0.0031 (6)
C11	0.0443 (8)	0.0376 (8)	0.0382 (8)	−0.0023 (6)	0.0146 (6)	0.0001 (6)
C7	0.0455 (9)	0.0397 (8)	0.0366 (7)	0.0017 (6)	0.0133 (6)	−0.0019 (6)
C16	0.0444 (8)	0.0530 (9)	0.0387 (8)	−0.0093 (7)	0.0152 (6)	−0.0092 (7)
C9	0.0433 (8)	0.0401 (8)	0.0386 (8)	0.0031 (6)	0.0121 (6)	−0.0018 (6)
C8	0.0418 (8)	0.0387 (8)	0.0374 (7)	0.0027 (6)	0.0121 (6)	−0.0009 (6)
C12	0.0489 (9)	0.0360 (8)	0.0382 (8)	−0.0027 (6)	0.0109 (6)	0.0002 (6)
C6	0.0474 (9)	0.0473 (9)	0.0405 (8)	0.0051 (7)	0.0117 (7)	−0.0017 (7)
C25	0.0466 (9)	0.0519 (9)	0.0459 (9)	−0.0101 (7)	0.0203 (7)	−0.0131 (7)
C17	0.0506 (10)	0.0646 (11)	0.0444 (9)	−0.0058 (8)	0.0143 (7)	−0.0030 (8)
C2	0.0461 (9)	0.0552 (9)	0.0449 (9)	0.0058 (7)	0.0135 (7)	−0.0019 (7)
C4	0.0436 (9)	0.0660 (11)	0.0511 (10)	−0.0024 (8)	0.0034 (7)	0.0071 (8)
C10	0.0495 (10)	0.0643 (11)	0.0533 (10)	0.0137 (8)	0.0139 (8)	−0.0075 (8)
C18	0.0556 (11)	0.0919 (15)	0.0382 (9)	−0.0157 (10)	0.0110 (8)	−0.0029 (9)
C24	0.0626 (11)	0.0551 (10)	0.0510 (10)	0.0013 (8)	0.0254 (8)	−0.0096 (8)
C5	0.0580 (10)	0.0540 (10)	0.0406 (8)	−0.0018 (8)	0.0055 (7)	−0.0039 (7)
C3	0.0446 (10)	0.0681 (11)	0.0546 (10)	0.0086 (8)	0.0131 (8)	0.0036 (9)
C19	0.0683 (12)	0.0818 (13)	0.0472 (10)	−0.0234 (10)	0.0239 (9)	−0.0240 (9)
C13	0.0486 (10)	0.0526 (10)	0.0580 (10)	−0.0104 (8)	0.0114 (8)	0.0019 (8)
C21	0.0903 (15)	0.0718 (13)	0.0745 (14)	−0.0120 (11)	0.0437 (12)	−0.0355 (11)
C20	0.0601 (11)	0.0620 (11)	0.0517 (10)	−0.0186 (8)	0.0260 (8)	−0.0205 (8)
C15	0.0667 (12)	0.0540 (10)	0.0559 (10)	0.0097 (9)	−0.0062 (9)	0.0075 (8)
C14	0.0471 (10)	0.0720 (12)	0.0595 (11)	0.0019 (9)	0.0053 (8)	0.0021 (9)
C23	0.0805 (14)	0.0602 (11)	0.0721 (12)	0.0112 (10)	0.0333 (11)	−0.0028 (10)
C22	0.1020 (17)	0.0592 (12)	0.0900 (16)	0.0080 (11)	0.0449 (13)	−0.0201 (11)

### Geometric parameters (Å, °)

**Table:**

O1—C7	1.2483 (17)	C2—H2	0.9300
N2—C9	1.3074 (18)	C4—C3	1.377 (2)
N2—N1	1.4062 (16)	C4—C5	1.377 (2)
N1—C7	1.3787 (18)	C4—H4	0.9300
N1—C1	1.4181 (18)	C10—H10A	0.9600
N3—C11	1.3388 (19)	C10—H10B	0.9600
N3—C16	1.4251 (18)	C10—H10C	0.9600
N3—H3A	0.890 (17)	C18—C19	1.356 (3)
O2—C15	1.363 (2)	C18—H18	0.9300
O2—C12	1.3677 (17)	C24—C23	1.361 (2)
C1—C6	1.387 (2)	C24—H24	0.9300
C1—C2	1.390 (2)	C5—H5	0.9300
C11—C8	1.3928 (19)	C3—H3	0.9300
C11—C12	1.461 (2)	C19—C20	1.410 (3)
C7—C8	1.444 (2)	C19—H19	0.9300
C16—C17	1.370 (2)	C13—C14	1.421 (2)
C16—C25	1.430 (2)	C13—H13	0.9300
C9—C8	1.439 (2)	C21—C22	1.356 (3)
C9—C10	1.491 (2)	C21—C20	1.420 (3)
C12—C13	1.339 (2)	C21—H21	0.9300
C6—C5	1.380 (2)	C15—C14	1.316 (3)
C6—H6	0.9300	C15—H15	0.9300
C25—C24	1.414 (2)	C14—H14	0.9300
C25—C20	1.425 (2)	C23—C22	1.397 (3)
C17—C18	1.411 (2)	C23—H23	0.9300
C17—H17	0.9300	C22—H22	0.9300
C2—C3	1.382 (2)		
			
C9—N2—N1	106.92 (11)	C5—C4—H4	120.6
C7—N1—N2	111.50 (11)	C9—C10—H10A	109.5
C7—N1—C1	129.74 (12)	C9—C10—H10B	109.5
N2—N1—C1	118.76 (11)	H10A—C10—H10B	109.5
C11—N3—C16	132.27 (14)	C9—C10—H10C	109.5
C11—N3—H3A	111.1 (11)	H10A—C10—H10C	109.5
C16—N3—H3A	114.8 (11)	H10B—C10—H10C	109.5
C15—O2—C12	106.02 (13)	C19—C18—C17	120.98 (17)
C6—C1—C2	119.41 (14)	C19—C18—H18	119.5
C6—C1—N1	119.62 (13)	C17—C18—H18	119.5
C2—C1—N1	120.92 (13)	C23—C24—C25	121.31 (16)
N3—C11—C8	117.99 (13)	C23—C24—H24	119.3
N3—C11—C12	120.57 (13)	C25—C24—H24	119.3
C8—C11—C12	121.43 (13)	C4—C5—C6	121.02 (16)
O1—C7—N1	126.48 (13)	C4—C5—H5	119.5
O1—C7—C8	128.64 (13)	C6—C5—H5	119.5
N1—C7—C8	104.88 (12)	C4—C3—C2	121.14 (16)
C17—C16—N3	123.69 (15)	C4—C3—H3	119.4
C17—C16—C25	120.68 (14)	C2—C3—H3	119.4
N3—C16—C25	115.34 (13)	C18—C19—C20	120.90 (16)
N2—C9—C8	111.25 (13)	C18—C19—H19	119.6
N2—C9—C10	119.01 (13)	C20—C19—H19	119.6
C8—C9—C10	129.61 (13)	C12—C13—C14	106.28 (15)
C11—C8—C9	132.15 (13)	C12—C13—H13	126.9
C11—C8—C7	122.51 (13)	C14—C13—H13	126.9
C9—C8—C7	105.33 (12)	C22—C21—C20	121.29 (17)
C13—C12—O2	109.92 (13)	C22—C21—H21	119.4
C13—C12—C11	134.89 (14)	C20—C21—H21	119.4
O2—C12—C11	115.19 (13)	C19—C20—C21	122.45 (16)
C5—C6—C1	119.89 (15)	C19—C20—C25	119.14 (17)
C5—C6—H6	120.1	C21—C20—C25	118.41 (17)
C1—C6—H6	120.1	C14—C15—O2	110.79 (15)
C24—C25—C20	118.23 (15)	C14—C15—H15	124.6
C24—C25—C16	123.42 (14)	O2—C15—H15	124.6
C20—C25—C16	118.34 (15)	C15—C14—C13	106.99 (16)
C16—C17—C18	119.84 (17)	C15—C14—H14	126.5
C16—C17—H17	120.1	C13—C14—H14	126.5
C18—C17—H17	120.1	C24—C23—C22	120.46 (19)
C3—C2—C1	119.63 (15)	C24—C23—H23	119.8
C3—C2—H2	120.2	C22—C23—H23	119.8
C1—C2—H2	120.2	C21—C22—C23	120.26 (19)
C3—C4—C5	118.90 (15)	C21—C22—H22	119.9
C3—C4—H4	120.6	C23—C22—H22	119.9
			
C9—N2—N1—C7	2.05 (16)	C2—C1—C6—C5	−1.3 (2)
C9—N2—N1—C1	−177.32 (12)	N1—C1—C6—C5	176.20 (14)
C7—N1—C1—C6	175.47 (14)	C17—C16—C25—C24	178.23 (15)
N2—N1—C1—C6	−5.3 (2)	N3—C16—C25—C24	−7.8 (2)
C7—N1—C1—C2	−7.0 (2)	C17—C16—C25—C20	−2.6 (2)
N2—N1—C1—C2	172.21 (13)	N3—C16—C25—C20	171.36 (14)
C16—N3—C11—C8	162.29 (15)	N3—C16—C17—C18	−173.62 (14)
C16—N3—C11—C12	−16.9 (3)	C25—C16—C17—C18	−0.1 (2)
N2—N1—C7—O1	176.89 (14)	C6—C1—C2—C3	1.1 (2)
C1—N1—C7—O1	−3.8 (3)	N1—C1—C2—C3	−176.36 (14)
N2—N1—C7—C8	−3.45 (15)	C16—C17—C18—C19	2.9 (3)
C1—N1—C7—C8	175.84 (13)	C20—C25—C24—C23	−1.0 (3)
C11—N3—C16—C17	−20.6 (3)	C16—C25—C24—C23	178.16 (17)
C11—N3—C16—C25	165.61 (16)	C3—C4—C5—C6	0.0 (3)
N1—N2—C9—C8	0.31 (17)	C1—C6—C5—C4	0.8 (2)
N1—N2—C9—C10	−175.88 (13)	C5—C4—C3—C2	−0.2 (3)
N3—C11—C8—C9	169.80 (15)	C1—C2—C3—C4	−0.4 (3)
C12—C11—C8—C9	−11.0 (3)	C17—C18—C19—C20	−2.8 (3)
N3—C11—C8—C7	−11.4 (2)	O2—C12—C13—C14	0.73 (19)
C12—C11—C8—C7	167.75 (14)	C11—C12—C13—C14	−179.64 (17)
N2—C9—C8—C11	176.57 (15)	C18—C19—C20—C21	179.81 (18)
C10—C9—C8—C11	−7.8 (3)	C18—C19—C20—C25	−0.1 (3)
N2—C9—C8—C7	−2.38 (17)	C22—C21—C20—C19	178.53 (19)
C10—C9—C8—C7	173.29 (16)	C22—C21—C20—C25	−1.6 (3)
O1—C7—C8—C11	4.0 (2)	C24—C25—C20—C19	−178.08 (15)
N1—C7—C8—C11	−175.66 (13)	C16—C25—C20—C19	2.7 (2)
O1—C7—C8—C9	−176.93 (16)	C24—C25—C20—C21	2.0 (2)
N1—C7—C8—C9	3.42 (15)	C16—C25—C20—C21	−177.14 (16)
C15—O2—C12—C13	−0.65 (19)	C12—O2—C15—C14	0.3 (2)
C15—O2—C12—C11	179.64 (14)	O2—C15—C14—C13	0.1 (2)
N3—C11—C12—C13	−57.2 (3)	C12—C13—C14—C15	−0.5 (2)
C8—C11—C12—C13	123.6 (2)	C25—C24—C23—C22	−0.6 (3)
N3—C11—C12—O2	122.37 (15)	C20—C21—C22—C23	0.0 (3)
C8—C11—C12—O2	−56.76 (19)	C24—C23—C22—C21	1.1 (3)

### Hydrogen-bond geometry (Å, °)

**Table:**

*D*—H···*A*	*D*—H	H···*A*	*D*···*A*	*D*—H···*A*
N3—H3A···O1	0.890 (17)	1.930 (17)	2.7030 (17)	144.3 (16)

## References

[bb1] Arıcı, C., Tahir, M. N., Ülkü, D. & Atakol, O. (1999). *Acta Cryst.* C**55**, 1691–1692. 10.1107/s0108270100009008 11077274

[bb2] Bruker (2005). *SMART* and *SAINT* Bruker AXS Inc., Madison, Wisconsin, USA.

[bb3] Chai, H., Liu, G. F., Liu, L. & Jia, D. Z. (2005). *Chin. J. Struct. Chem.* **24**, 1091–1095.

[bb4] Dong, X.-C., Liu, F.-C. & Zhao, Y.-L. (1983). *Acta Chim. Sin.* **41**, 848–852.

[bb5] Holzer, W., Mereiter, K. & Plagens, B. (1999). *Heterocycles*, **50**, 799–818.

[bb6] Jensen, B. S. (1959). *Acta Chem. Scand.* **13**, 1668–1670.

[bb13] Li, J.-Z., Li, G. & Yu, W.-J. (2000). *J. Rare Earth*, **18**, 233–236.

[bb7] Lü, X. Q., Bao, F., Kang, B. S., Wu, Q., Liu, H. Q. & Zhu, F. M. (2006). *J. Organomet. Chem.* **691**, 821–828.

[bb8] Mehrotra, R. C., Bohra, R. & Gaur, D. P. (1978). *Metal β-diketonates and Allied Derivatives* New York: Academic Press.

[bb9] Peng, B., Liu, G., Liu, L., Jia, D. & Yu, K. (2004). *J. Mol. Struct* **692**, 217–222.

[bb10] Sheldrick, G. M. (2005). *SADABS* University of Göttingen, Germany.

[bb11] Sheldrick, G. M. (2008). *Acta Cryst.* A**64**, 112–122. 10.1107/S0108767307043930 18156677

[bb12] Uzoukwu, A. B., Al–Juaid, S. S., Hitchcock, P. B. & Smith, J. D. (1993). *Polyhedron*, **12**, 2719–2724.

