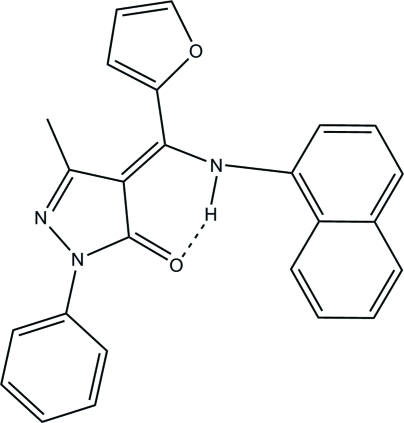# 4-[(*Z*)-(2-Fur­yl)(2-naphthyl­amino)methyl­ene)]-3-methyl-1-phenyl-1*H*-pyrazol-5(4*H*)-one. Corrigendum

**DOI:** 10.1107/S1600536810002527

**Published:** 2010-01-30

**Authors:** Jing Li, Jin-Zhou Li, Jing-Qi Li, Heng-Qiang Zhang, Jia-Min Li

**Affiliations:** a College of Chemistry and Chemical Engineering, Harbin Normal University, Harbin 150025, People’s Republic of China

## Abstract

The title of the paper by Li, Li, Li, Zhang & Li [*Acta Cryst.* (2009), E**65**, o1824] is corrected.

## Abstract 2

Corrigendum to *Acta Cryst.* (2009), E**65**, o1824.

In the paper by Li *et al.* (2009) ▶, the chemical name given in the *Title* should be ‘4-[(*Z*)-(2-Fur­yl)(1-naphthyl­amino)methyl­ene]-3-methyl-1-phenyl-1*H*-pyrazol-5(4*H*)-one’. The revised scheme is shown below.